# Oxidative Stress: The Role of Estrogen and Progesterone

**DOI:** 10.3390/jcm12237304

**Published:** 2023-11-25

**Authors:** Angelo Cagnacci, Irene Gazzo, Sara Stigliani, Anna Maria Paoletti, Paola Anserini, Ambrogio Pietro Londero, Anjeza Xholli

**Affiliations:** 1 Obstetrics and Gynecology Teaching Unit, IRCCS Ospedale Policlinico San Martino, 16132 Genoa, Italy; irigazzo@libero.it (I.G.); paola.anserini@hsanmartino.it (P.A.); anj160583@yahoo.it (A.X.); 2 Department of Neuroscience, Rehabilitation, Ophthalmology, Genetics, Maternal and Infant Health, University of Genoa, 16132 Genova, Italy; 3 Physiopathology of Human Reproduction, IRCCS Ospedale Policlinico San Martino, 16132 Genoa, Italy; 4 Obstetrics and Gynecology Teaching Units, Azienda Ospedaliera Universitaria di Cagliari, 09124 Cagliari, Italy; gineca.annapaoletti@tiscali.it

**Keywords:** oxidative stress, ROS, antioxidant, ovarian stimulation, menstrual cycle, contraception, hormonal contraceptives, cardiovascular disease, cancer

## Abstract

The effect of estrogen and progesterone on oxidative status is not yet very clear, improvements and detrimental effects having been reported with the use of menopausal hormone therapy or hormonal contraceptives, respectively. In this study, we evaluated the role played by estrogen and progesterone separately, on the oxidative status of 32 women, 18 to 43 years old, by inducing high levels of estrogen and then adding high levels of progesterone. During a cycle of in vitro fertilization, blood samples were collected prior to gonadotrophin stimulation (low estradiol levels), on the day of oocyte retrieval (high levels of estrogen), and on the day of embryo transfer (high levels of estrogen and progesterone). Total blood levels of oxidants (FORT), antioxidants (FORD), and their ratio FORT/FORD were measured using a colorimetric method based on the Fenton reaction. Seven women measured their early morning body temperature at the same time points. FORT significantly decreased from the low- to the high-estrogen phase (*p* = 0.023) and increased from the high-estrogen to the high-estrogen–progesterone phase (*p* = 0.006). FORD showed an opposite but non-significant trend. The FORT/FORD ratio decreased from the low- to the high-estrogen phase (*p* = 0.0104) and increased from the high-estrogen to the high-estrogen –progesterone phase (*p* = 0.004). Body temperature (*n* = 7) decreased in the high-estrogen phase (*p* = 0.001) and increased from the high-estrogen to the high-estrogen–progesterone phase (*p* = 0.001). In the seven women, FORT (*p* = 0.009) and FORT/FORD (*p* = 0.0056) were linearly related to body temperature values. Our data show opposite effects of estrogen and progesterone on oxidative status. These effects seem to be related to the effect exerted on body temperature regulation.

## 1. Introduction

Oxidative stress represents an overproduction of reactive oxygen species, like hydroxyl radicals, peroxide radicals, and superoxide anions, which is not appropriately balanced by both enzymatic and nonenzymatic antioxidant defenses [1]. It negatively impacts fertility and plays a significant role in the pathogenesis of malignancies, neurological and cardiovascular degenerative diseases, and in general, in the aging processes [2,3,4,5].

The role played by gonadal steroids on oxidative stress is still unclear. Increased oxidative stress has been reported by several [6,7,8,9,10,11,12], but not all [13] studies, during the use of hormonal contraceptives supposedly due to a high-estrogen stimulus [12,14]. Vice–versa data obtained in postmenopausal women indicate that the increase in oxidative status is due to a lack of estrogen, and that estrogen administration decreases it [15,16]. The role played by the progestin stimulus has garnered little interest. Oxidative stress is associated with increased metabolic activity [4,17], and progesterone increases resting metabolic activity to induce an approximate 0.4 °C increase in body temperature [18,19,20]. This effect of progesterone was documented in the normal menstrual cycle when comparing values of body temperature of the follicular and luteal phases, in stimulated cycles, comparing temperature values of the high-estrogen and the high-estrogen –progesterone phases [18,19,20], or even during the administration of hormonal contraceptives [21,22]. In this study, we tried to disentangle the role played by estrogen and progesterone on oxidative status. To this end, the oxidative status of women undergoing a stimulated cycle for in vitro fertilization was evaluated during a low estrogen–progesterone stimulus, a high-estrogen stimulus, and a high-estrogen-plus-progesterone stimulus. Oxidative status was determined by measuring total blood oxidants (FORT), antioxidants (FORD), and their ratio FORT/FORD [6,13,23,24,25,26].

## 2. Materials and Methods

### 2.1. Ethics Committee

This study was approved by the independent ethics committee of Cagliari (No. PG 2020/7598, approved 25 March 2020). Each woman signed an informed consent form authorizing her participation in the study and the use of her data in scientific analyses and publications.

### 2.2. Patient Characteristics

Between September 2021 and February 2023, 32 women aged 18 to 43 years were recruited among those undergoing fertility treatments.

Exclusion criteria were smoking, daily consumption of alcohol exceeding the equivalent of a glass of wine with 11% alcohol volume, and the use of any nutritional supplements, antioxidants, or vitamins that could have interfered with oxidative stress.

### 2.3. Controlled Ovarian Stimulation Procedure

Women were managed in line with standard clinical practice. Infertility was diagnosed after at least 12 months of attempts to conceive, as defined by the World Health Organization.

All the included women started a controlled ovarian stimulation cycle following a protocol with the GnRH antagonist.

Controlled ovarian stimulation was performed, starting on day 2 of the menstrual cycle, via the administration of subcutaneous gonadotropins at a dose that was personalized daily based on the patient’s parameters (between 150 and 300 international units of Puregon, Oss, The Netherlands). From day 2 to oocyte pick-up, a blood sample was collected daily at 8 am to measure circulating estradiol and progesterone levels. An additional blood sample was collected at embryo transfer. Follicle growth was evaluated with a transvaginal ultrasound scan.

The GnRH antagonist (Ganirelix 0.25 mg, Fyremadel, Sun Pharmaceutical Industries Europe, Hoofddorp, The Netherlands) was administered after 5 to 7 days of gonadotrophin stimulation based on estrogen levels (>200 pg/mL) and ultrasonographic number and dimension of ovarian follicles (at least 3 follicles >11 mm or 1 leading follicle ≥13 mm). When the leading follicle(s) reached 18 mm in size, highly purified human chorionic gonadotropin 10,000 IU (Gonasi HP, IBSA Farmaceutici Italia, Lodi, Italy) was used to induce final oocyte maturation. Oocyte retrieval was performed 35–36 h later. In the case of ovarian hyperstimulation (i.e., the presence of more than 15 oocytes recovered at the time of pick-up), abdominal pain, and high levels of E2, oocytes were inseminated, and embryos or blastocysts were vitrified to be used in a subsequent transfer cycle; the cycle was suspended, and the patient excluded from the study.

After oocyte pick-up, to reinforce the luteal phase, patients started vaginal progesterone (Amelgen, Gedeon Richter Italia, Milano, Italy) 400 mcg twice a day.

### 2.4. Study Design

For each woman, age, medical and reproductive history, weight in kilograms, height in meters, body mass index (BMI, Kg/m^2^), the leading cause of infertility, smoking habit, and factors that may influence oxidative status were collected and recorded in an electronic database by a physician. Women were invited not to change their diet or to take supplements or vitamins during the ovarian stimulation cycle.

FORD, FORT, and FORT/FORD ratio were immediately evaluated in samples of capillary blood collected from a finger, at 8:00 in the morning and after overnight fasting. Oxidative status was determined at three time points: the second day of the menstrual cycle, prior to gonadotrophin stimulation (low estradiol levels); the day of oocyte pick-up (high estradiol levels), and the day of embryo transfer (high estradiol and progesterone levels).

### 2.5. Body Temperature Evaluation

Some patients were asked to measure their oral temperatures daily, at the time of wake-up, using an alcohol thermometer. Temperature, expressed in degrees Celsius, was reported on a chart from the first day of the cycle to the day following the embryo transfer. The value of body temperature representative of each of the 3 phases was considered the mean value of 3 days, from the day preceding to the one following the day of oxidative status evaluation.

### 2.6. Measurement of Hormones

Serum levels of anti-mullerian hormone (AMH) were measured using the Roche Elecsys AMH system (Elecsys, Roche Diagnostics, Indianapolis, IN, USA) within a range of 0.01–23 ng/mL, and a coefficient of variation (CV) of 1.0–1.6% which was 0.055–19 ng/mL. Serum estradiol was measured using the ACCESS immunoassay System (Beckman Coulter, Brea, CA, USA), with a range of 15–4704 pg/mL, and an intra-assay CV of 1.4–4.2%, which was 21–4033 pg/mL. Serum progesterone was measured using the ACCESS Immunoassay System (Beckman Coulter, Brea, CA, USA), with a range of 0.10–40.0 ng/mL, and an intra-assay CV of 6.11–11.19%, which was 1.26–22.6 ng/mL.

### 2.7. Measurement of Oxidative Status

The method of determining oxidative status has already been reported in several publications [13,23,24,25,26,27]. Reactive oxygen species were determined in 20 μL of capillary blood using the FORT test (FORM^®^, CR 2000, Callegari, Parma, Italy)—a colorimetric assay based on the capacity of transition metals to stimulate the breakdown of hydroperoxides (ROOH) into derivative radicals—within the Fenton reaction. The radical species interact with phenylenediamine derivative (2CrNH_2_), which forms a long-lasting, colored, radical cation that can be assayed using a spectrophotometer at 505 nm. The intensity of the color correlates directly with the abundance of radical compounds and, consequently, with the oxidative status of the sample, according to

R-OOH + Fe_2_+ → RO· + OH− + Fe_3_+

R-OOH + Fe_3_+ → ROO· + H+ + Fe_2_+

RO· + ROO· + 2CrNH2→ ROO− + RO− + [CrNH_2_+·]

The results are expressed as FORT units, whereby 1 FORT unit corresponds to 0.26 mg/L of H_2_O_2_. The method’s intra-assay and inter-assay coefficients of variation were 3.7% and 6.2%, respectively.

The FORD test measures the plasma antioxidant system using preformed, stable, colored radicals. It determines the decrease in absorbance, which is proportional to the blood antioxidant concentration of the sample, according to the Beer–Lambert law. In the presence of an acidic buffer (pH = 5.2) and a suitable oxidant (FeCl_3_), the chromogen—which contains 4-amino-N, N-diethylaniline sulfate—forms a stable and colored radical cation that is photometrically detectable at 505 nm. Antioxidant compounds in the sample reduce the radical cation of the chromogen, quenching the color and producing a discoloration of the solution in proportion to their concentration. The absorbance values obtained for the samples were compared with a standard curve obtained using Trolox (6-hydroxy-2,5,7,8-tetramethylchroman-2-carboxylic acid), a permeable cell derivative of vitamin E commonly used as an antioxidant [25,28]. FORD results are expressed as Trolox equivalents (mmol/L) using a calibration curve plotted with different amounts of standard Trolox that are stored on the dedicated instrument (FORM Plus, FORM ox and CR3000 series diagnostic analyzers, Callegari SpA, Catellani Group, Parma, Italy). All reagents were kept at room temperature. The ratio of FORT/FORD was used to define oxidative status.

### 2.8. Statistical Analysis

By setting the type I error at 0.05 and type II error at 0.2, 8 subjects were sufficient to document a within-group difference that was higher than the SD of the mean difference. The number of women enrolled was similar or higher than that reported in similar studies analyzing oxidative status in OC users, or in women during the menstrual phase [8,9,12,29,30,31,32]. Based on an a posteriori power analysis, it has been determined that the study achieved a minimum power level of 80% for an effect size of 0.5 by considering the participation of 32 patients.

An analysis of variance (ANOVA) for repeated measures, with subjects as replicates, was used to compare FORT, FORD, FORT/FORD, and oral temperature in the 3 different moments of the in vitro fertilization cycle. When significant, the post-hoc test of Fisher was used to compare the different groups. Because age may have an influence on oxidative status, we also tested whether age, introduced as covariate, may influence the results. Simple regression analysis was used to correlate FORT, FORD, or FORT/FORD with body temperature values. Analyses were performed using the StatView 5.1 statistical package (1985 SAS Institute, Mountain View, CA, USA). All results are expressed as means and standard deviations. A *p*-value <0.05 is considered statistically significant.

## 3. Results

### 3.1. Clinical Characteristics

The women’s characteristics are reported in Table 1.

The primary cause of infertility was idiopathic for 10, a male factor for 6, and a female factor for 16 other women (Table 1). Most women were non-smokers (75%), performed mild physical activity, and did not use or used a limited amount of alcohol.

### 3.2. Hormone Levels

Controlled ovarian stimulation had an average duration of 11.1 ± 1.55 days, and oocyte retrieval was scheduled 13.1 ± 1.55 days after initiating the gonadotrophin stimulation. The mean estradiol levels were 56.9 ± 28.8 pg/mL on the second day of the menstrual cycle, 1973.5 ± 1013.9 pg/mL at the time of oocyte pick-up, and 2133.1 ± 1205.3 at the time of the embryo transfer (*p* = 0.001). Within the 3 days, the corresponding levels of progesterone were 0.25 ± 0.30 ng/mL, 1.11 ± 0.81 ng/mL, and >40 ng/mL, respectively (*p* = 0.001).

### 3.3. Body Temperature Values

Only seven subjects appropriately and completely monitored their body temperature. In these women, body temperature decreased from the low-estrogen phase (the second day of the cycle) to the high-estrogen phase (time of oocyte pick-up) from 36.31 ± 0.42 to 35.93 ± 0.41 °C (*p* = 0.001). Vice versa, body temperature significantly increased in the estrogen–progesterone phase (the day of embryo transfer) to values of 36.72 ± 0.32 °C (*p* = 0.001) (Table 2).

### 3.4. Oxidative Status

Oxidative factors, evaluated by FORT, significantly changed (*p* = 0.013) they decreased from the low- to the high-estrogen phase (3.92 ± 1.02 vs. 3.36 ± 0.91, *p* = 0.023) to increase in the estrogen–progesterone phase (3.36 ± 0.91 vs. 4.05 ± 1.01, *p* = 0.006) (Table 2).

A non-significative trend was observed for antioxidant defenses. FORD tended to increase from the low- to the high-estrogen phase (0.93 ± 0.15 vs. 1.02 ± 0.25, *p* = 0.085) to decrease in the high estrogen-progesterone phase (1.02 ± 0.25 vs. 0.92 ± 0.14, *p* = 0.052) (Table 2).

The ratio between oxidant and antioxidant factors (FORT/FORD) showed a significant variation (*p* = 0.0096) among the three different phases, decreasing from the low- to the high-estrogen phase (4.39 ± 1.58 vs. 3.49 ± 1.24, *p* = 0.01) and increasing in the estrogen–progesterone phase (3.49 ± 1.24 vs. 4.53 ± 1.39, *p* = 0.004) (Table 2). Age when introduced into the analysis was not related to oxidative status and its modifications.

### 3.5. Relation between Oxidative Status and Body Temperature

**Figure 1:**
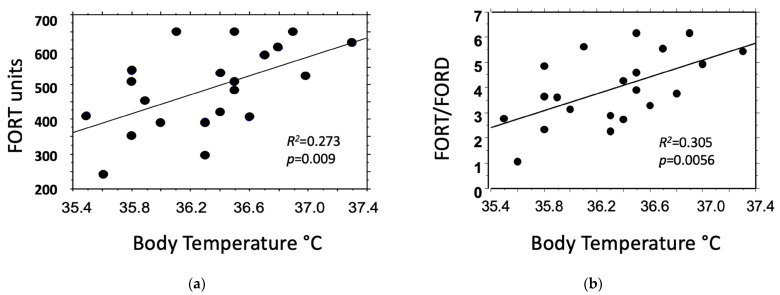
Linear regression analysis between body temperature values measured in seven women during the three phases of the stimulated cycle and (**a**) blood oxidants (FORT), or (**b**) the ratio of blood oxidants/antioxidants (FORT/FORD).

Upon linear regression analysis, body temperature was negatively related to FORD (*p* = 0.025) and positively related to FORT (*p* = 0.009) and the FORT/FORD ratio (*p* = 0.0056) (Table 3 and Figure 1).

## 4. Discussion

Oxidative status was evaluated under low estrogen–progesterone levels, supraphysiological estrogen, and similar supraphysiological levels of estrogen plus supraphysiological levels of progesterone. The data indicate that oxidative status, as measured by FORT or the FORT/FORD ratio, decreases in the presence of supraphysiological levels of estrogen, and from this condition, it is increased by the contemporaneous presence of supraphysiological levels of progesterone. The data on estrogen fit with evidence obtained in postmenopausal women in which estrogen administration decreases oxidative stress, upregulating antioxidant and longevity-related genes [33] and protecting against oxidative stress-related diseases, like cardiovascular disease [34]. Data obtained in 9 [29] 12 [30] and 20 [31] women studied during the menstrual cycle reported inconsistent results. No modifications in oxidative status were observed in two studies [29,30], while in the third study, oxidants increased from the early to the late follicular phase [30]. The authors hypothesized a pro-oxidant effect of estrogen, but maximal levels of oxidants were also observed in the luteal phase, with the concomitant presence of estrogen and progesterone [31]. Antioxidants and the oxidant/antioxidant ratio were not evaluated [31].

The capability of progesterone to increase oxidative stress was not unexpected, since progesterone enhances protein catabolism [35,36], and increases oxygen-reactive species, overloading their paths of elimination [37].

Oxidative stress is a phenomenon strictly related to energy expenditure, increasing during exercise or temperature elevation [4,17]. Estrogen and progesterone exert opposite effects on temperature regulation. Estrogen decreases body temperature and energy expenditure, and progesterone does the opposite [18,38]. In the subset of subjects who measured it, body temperature was linearly related to oxidative status, with low and high body temperature values being associated with low and high oxidative stress, respectively.

Because the combination of estrogen and progestin in hormonal contraceptives increases body temperature [21,22], it may also increase oxidative status, as previously reported [3,6,8,9,10,11,12,30,32].

### Strength and Limitations

This study has several limitations. We did not specifically measure oxidant molecules, antioxidant substances, or enzymes, but instead the oxidative balance. Free oxygen radicals are highly complex to evaluate in vivo due to their rapid metabolism [39], and our colorimetric method has been extensively used in previous studies [13,23,24,25,26,27]. The study was performed in a single center on Caucasian women. We clearly documented an association between different endocrine environments and oxidative stress in the 32 enrolled women, with opposite effects exerted by estrogen and progesterone. Progesterone levels were measured in the general laboratory of our hospital, with a commercially available kit whose upper limit was 40 ng/mL. Data above this limit were not available. Whether appropriate for clinical purposes, this did not allow for the calculation of the progesterone/estradiol ratio, and of its eventual relation with oxidative status, as previously reported for body temperature [18]. Additional studies are necessary to explore this possibility. However, this relation was indirectly supported by the fact that in seven women, oxidative status showed a correlation with body temperature. In these women, probably by chance, the oxidative status of the high-estrogen–high-progesterone phase was also higher than in the low-estrogen–low-progesterone phase. The result was not so clear when considering the entire group of women, suggesting that in some women the balance between progesterone and estradiol, and consequently on oxidative status, of the high-estrogen–high-progesterone phase was similar to that of the low-estrogen and low-progesterone phase. The study tested supraphysiological levels of estradiol and progesterone. Accordingly, it is unclear how these data can be applied to physiological conditions. A short-lasting decrease in body temperature at the preovulatory estradiol peak and a sustained increase in body temperature in the luteal phase have been clearly documented [18,19,20,40,41], but modifications in oxidative stress were not consistently observed during the menstrual cycle [29,30,31]. Because our study was performed on endogenous estrogen and natural progesterone, the data do not necessarily apply to different estrogen or progestin molecules. An increase in body temperature has been commonly reported during progestin administration [21,22,41,42], except for dihydrogesterone [42]. Whether a neutral effect on body temperature, and likely on oxidative status, may have a different impact on women’s health deserves further investigation. Interestingly, epidemiological data show that in contrast to other progestins, dihydrogesterone administration does not increase the risk of breast cancer [43].

## 5. Conclusions

In conclusion, our data show that an elevated estrogen stimulus decreases oxidative status in young women undergoing a stimulated cycle for infertility. In this condition, the adjunct of an elevated progesterone stimulus increases oxidative stress. These effects seem to be related to a concomitant decrease and increase in body temperature, respectively. These data were obtained in a limited number of infertile women, and should be confirmed by more extensive studies performed in different populations of women.

## Figures and Tables

**Table 1:** Characteristics of the 32 enrolled women.

Age (yrs)	37.09 ± 4.06
BMI (kg/m^2^)	24.03 ± 4.91
AMH (ng/mL)	2.52 ± 1.87
Cause of infertility (n)	
Idiopathic	10
Male Factor	6
Female Factor	16
Tubaric	6
Low Reserve	4
PCOS	4
Myomas	2
Smoking (*n*)	
Smokers	4
Ex-Smokers	4
Non-Smokers	24
Physical Activity (h/week)	1.87 ± 1.89
Use of Alcohol (glass/week)	0.5 ± 0.3
None	10
0 to ≤2	18
>2	3

When necessary, data are expressed as mean ± SD.

**Table 2:** Mean (±SD) values of body temperature obtained in 7 women, and of oxidants (FORT), antioxidants (FORD), and their ratio (FORT/FORD) obtained in 32 women at three time points of a stimulated cycle for assisted reproduction.

	A 2nd Day	B Pick-Up	C ET	A vs. B *p* Value	A vs. C *p* Value	B vs. C *p* Value
Body Temperature * (°C)	36.3 ± 0.4	35.9 ± 0.4	36.7 ± 0.3	0.001	0.001	0.001
FORT (units)	3.92 ± 1.020	3.36 ± 0.91	4.05 ± 1.01	0.023	0.584	0.006
FORD (units)	0.93 ± 0.15	1.02 ± 0.25	0.92 ± 0.14	0.085	0.783	0.052
FORT/FORD	4.39 ± 1.58	3.49 ± 1.24	4.53 ± 1.39	0.01	0.682	0.004

* Data obtained from only seven women. ET: embryo transfer; FORT: oxidants in blood; FORD: antioxidants in blood.

**Table 3:** Simple linear regression analysis between body temperature (BT), and blood oxidants (FORT), antioxidants (FORD), or their ratio (FORT/FORD) in the seven subjects who measured body temperature during the stimulation cycle.

Body Temperature (°C) vs.	Coefficient of Regression	95% Confidence Interval	*R* ^2^	*p*-Value
FORT units	1.031	0.291, 1.771	0.273	0.009
FORD units	−0.225	−0.417, −0.033	0.201	0.025
FORT/FORD	1.685	0.556, 2.814	0.305	0.0056

## Data Availability

The data that support the findings of this study are available, but restrictions apply to the availability of these data, which were used under license for the current study and so are not publicly available. However, data are available from the authors upon reasonable request and with the permission of the Internal Review Board.
